# New Cretaceous Lacewings in a Transitional Lineage of Myrmeleontoidea and Their Phylogenetic Implications †

**DOI:** 10.3390/insects13050429

**Published:** 2022-05-05

**Authors:** Xiumei Lu, Chunpeng Xu, Xingyue Liu

**Affiliations:** 1 Institute of Ecological and Environmental Protection, Shanghai Academy of Agricultural Sciences, Shanghai 201403, China; lxm2361892563@126.com; 2 State Key Laboratory of Palaeobiology and Stratigraphy, Nanjing Institute of Geology and Palaeontology and Center for Excellence in Life and Paleoenvironment, Chinese Academy of Sciences, 39 East Beijing Road, Nanjing 210008, China; cpxu@nigpas.ac.cn; 3 University of Chinese Academy of Sciences, Beijing 100049, China; 4 Department of Entomology, China Agricultural University, Beijing 100193, China

**Keywords:** Cratosmylidae, Babinskaiidae, new taxa, phylogeny, Cretaceous, Kachin amber

## Abstract

**Simple Summary:**

The Cretaceous myrmeleontoids (antlions, spoon-winged lacewings, split-footed lacewings, etc.), as one of the diverse neuropteran groups, are valuable for understanding the early evolution of Myrmeleontoidea. Here, two new species individually belonging to the extinct families Cratosmylidae and Babinskaiidae are described from the mid-Cretaceous Kachin amber of Myanmar. The morphology-based phylogeny of Myrmeleontoidea herein inferred recovered the positions of these new taxa but questioned the familial status of Cratosmylidae. The new finding also highlights the Gondwanan origin of the lacewing paleofauna from the mid-Cretaceous of northern Myanmar.

**Abstract:**

The extinct neuropteran families Cratosmylidae and Babinskaiidae hitherto only known from the Cretaceous represent the transitional lineage between Nymphidae and advanced myrmeleontoids (e.g., Nemopteridae and Myrmeleontidae) in the superfamily Myrmeleontoidea. Here, we describe two new species, which respectively belong to Cratosmylidae and Babinskaiidae, namely, *Araripenymphes burmanus* sp. nov. and *Paradoxoleon chenruii* gen. et sp. nov., from the mid-Cretaceous Kachin amber of Myanmar. Cratosmylidae, which was previously only recorded from the Lower Cretaceous of Brazil (Crato Formation), is first reported from the mid-Cretaceous Kachin amber of Myanmar, and the co-occurrence of *Araripenymphes* Menon, Martins-Neto and Martill, 2005 across South America and Asia further documents the Gondwanan origin of the northern Myanmar amber lacewing paleofauna. The first finding of a deeply bifurcated forewing MP with two free branches in Babinskaiidae (viz., *Paradoxoleon chenruii* gen. et sp. nov.) highlights the morphological diversity of this extinct family. The phylogenetic positions of *Araripenymphes burmanus* sp. nov. and *Paradoxoleon chenruii* gen. et sp. nov. were recovered on the basis of a morphology-based phylogenetic analysis, and the monophyly of Cratosmylidae + Babinskaiidae was corroborated. Given the paraphyly of Cratosmylidae, its familial status is discussed.

## 1. Introduction

The superfamily Myrmeleontoidea is one of the most species-rich group of Neuroptera, with around 2300 extant species in three families, i.e., Nymphidae (split-footed lacewings), Nemopteridae (thread-winged or spoon-winged lacewings), and Myrmeleontidae (antlions and owlflies) [1,2,3,4]. The superfamily as a whole has a worldwide distribution, while Nymphidae only ranges in the Australasian region and Nemopteridae does not occur from the North America [5,6]. The morphological diversity of Myrmeleontoidea is remarkable in having some species with elongated mouthparts for pollen feeding, wing eyespots for threating display, long-neck-like larval pronotum for predation, and thorny larval body for camouflage [6,7].

The origin of Myrmeleontoidea dates back at least to the Middle Jurassic according to the oldest fossil record, a stem Myrmeleontidae, i.e., *Choromyrmeleon othneius* Ren and Guo, 1996 and *C. aspoeckorum* Ren and Engel, 2008 [8,9], but it may be older, probably during the Triassic, as estimated by molecular dating [10,11]. Hitherto, Myrmeleontoidea have been recorded with around 95 fossil species, and most of them are from the Cretaceous, constituting a diverse paleofauna, which includes the species of not only all extant families (Nymphidae, Nemopteridae, and Myrmeleontidae) but also of three extinct families (i.e., Cratosmylidae, Babinskaiidae, and Palaeoleontidae) [3,4,12,13,14,15,16,17]. Babinskaiidae, together with Cratosmylidae, represent a transitional lineage between Nymphidae and advanced Myrmeleontoidea (i.e., Nemopteridae and Myrmeleontidae), being of a phylogenetic significance for understanding the evolution of the Myrmeleontoidea [18].

Babinskaiidae is a well-known extinct family of Myrmeleontoidea, currently with 17 species in 13 genera, most of which are recorded from the Lower Cretaceous (Aptain) of Brazil and the mid-Cretaceous Kachin amber of Myanmar [18,19,20,21,22,23,24,25], Table S1. On the basis of previous knowledge, the adults of Babinskaiidae are characterized by the filiform antennae, the presence of trichosors, the origin of RP + MA far distal to wing base, the presence of presectoral crossveins in both fore- and hind wings, the forewing MP simple, and the reduction of hind wing A2 and A3 veins. Cratosmylidae, however, is little known in the aspects of morphology, species diversity, and phylogenetic status. This family was first established by Makarkin et al. [23] on the basis of two extinct genera: *Araripenymphes* Menon, Martins-Neto and Martill, 2005 and *Cratosmylus* Myskowiak, Escuillié and Nel, 2015 from the Lower Cretaceous of Brazil, which were once placed in Nymphidae [13,26]. This family is characterized by the presence of trichosors, the origin of RP + MA near wing base, the presence of presectoral crossveins in both fore- and hind wings, and the forewing MP deeply forked. In the myrmeleontoid phylogeny inferred by Lu et al. [18], the two cratosmylid genera were assigned into a monophylum with Babinskaiidae, but the monophyly of this family was not recovered.

The mid-Cretaceous Kachin amber from northern Myanmar has received extensive study in recent years, as it preserves a diverse biota of the Cretaceous Neuroptera, which provides valuable direct evidence for the understanding of the phylogeny, biogeography, ecology, and early evolution of the lacewings. Here, we describe a new species of the cratosmylid genus *Araripenymphes* and a new genus and species of Babinskaiidae from the mid-Cretaceous Kachin amber, northern Myamar, namely, *Araripenymphpes burmanus* sp. nov. and *Paradoxoleon chenruii* gen. et sp. nov. Cratosmylidae, first recorded from the mid-Cretaceous Kachin amber of Myanmar. The new babinskaiid genus possesses a strongly elongated veinlet interlinked with the forewing presectoral crossvein and the RP + MA, as well as a deeply diverged forewing MP, which have yet been found in Babinskaiidae. A phylogenetic analysis based on an updated morphological dataset of Lu et al. [18] was performed to infer the phylogenetic positions of these two new taxa. The results provide new insight into the morphological diversity, phylogeny, and evolutionary history of Myrmeleontoidea.

## 2. Materials and Method

### 2.1. Taxonomy

**Figure 1:**
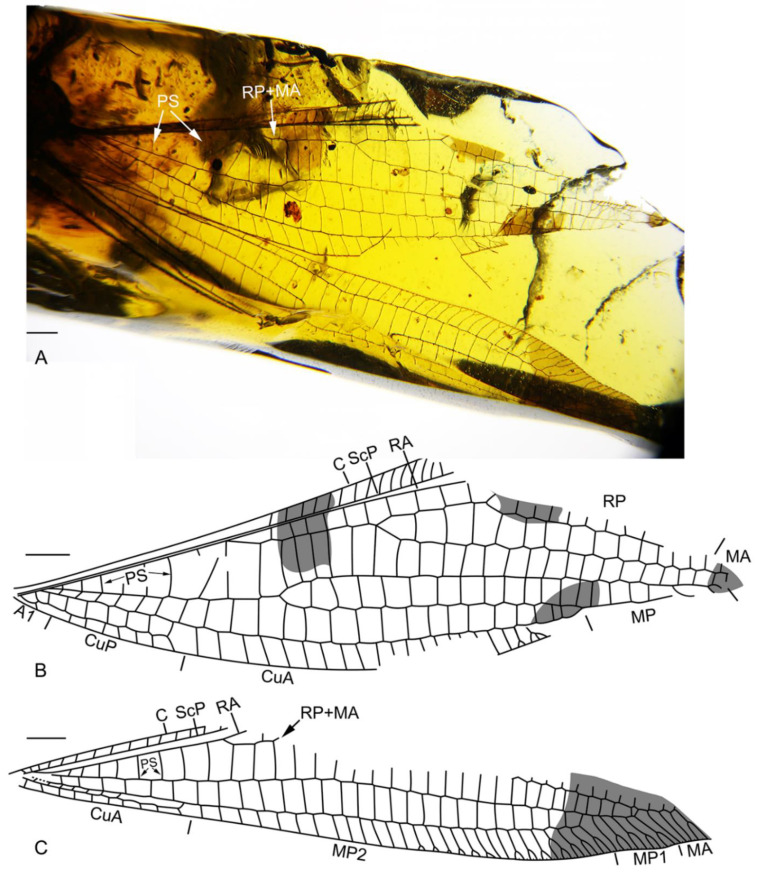
*Araripenymphes burmanus* sp. nov., holotype: NIGP180156. (**A**) Photographs of fore- and hind wings; (**B**) drawing of forewing; (**C**) drawing of hind wing. Scale bar = 1.0 mm.

The amber specimens described herein are from the Hukawng Valley, Tanai Township, Myitkyina District, Kachin State, Myanmar ([27]: figure 1). The age of this deposit is dated to be ≈99 million years (the earliest Cenomanian) by U-Pb dating of zircons from the volcaniclastic matrix of the amber [28].

The type specimens of *Araripenymphes burmanus* sp. nov. and *Paradoxoleon chenruii* gen. et sp. nov. are separately deposited in the Nanjing Institute of Geology and Palaeontology (NIGP), Chinese Academy of Sciences, Nanjing, China, and the Time Capsule Museum of Insect Amber, Sanming, Fujian, China.

Photographs and drawings were taken or made using a Leica M125C microscope system connecting with a Canon EOS 5D Mark IV camera system (Canon Inc., Japan). The figures were prepared with Adobe Photoshop CC 2017. Terminology of wing venation generally follows Aspöck et al. [29] and Martins-Neto [30]. Terminology of genitalia follows Aspöck, U. and Aspöck, H. [31].

Abbreviations used for wing veins are as follows: A, anal vein; C, costa; Cu, cubitus; CuA, cubitus anterior; MP, media posterior; R, radius; RA, radius anterior; RP, radius posterior; ScA, subcosta anterior; ScP, subcosta posterior; ps, presectoral crossveins (i.e., r-mp crossveins).

### 2.2. Phylogenetic Analysis

Aiming to recover the phylogenetic position of the two new myrmeleontoid taxa, a phylogenetic analysis of Myrmeleontoidea was performed on the basis of the morphological dataset from Lu et al. [18], with a newly described babinskaiid genus (i.e., *Paraneliana* Jouault and Nel, 2021) and the two new taxa added, with the character coding updated.

The present character comprises a total of 54 adult characters (see Note S1). Unknown characters were coded as “?”, while inapplicable characters were coded as “-”. The data matrix is also given in Table S2. All characters were treated as unordered and with equal weight. We analyzed the dataset using TNT v1.5 [32] with an initial traditional search (starting trees: 100 repls, TBR, trees to save per replication: 10). Bremer support values and bootstrap values were calculated with the function implemented in TNT (setting for Bremer support values calculation: TBR from existing trees, retain trees suboptimal by 10 steps; setting for bootstrap values calculation: traditional search, number of replicates: 1000). Character states were mapped on the strict consensus tree (MPT) using WinClada ver. 1.00.08 [33], showing only unambiguous changes.

## 3. Results

### 3.1. Systematic Paleontology

Class Insecta Linnaeus, 1758

Order Neuroptera Linnaeus, 1758

Superfamily Myrmeleontoidea Latreille, 1802

Family Cratosmylidae Makarkin, Heads and Wedmann, 2017

Type genus: *Cratosmylus* Myskowiak, Escuillié and Nel, 2015

Diagnosis. (1) Forewing broadly ovoid or narrowly elongated; (2) trichosors present along wing margin of both fore- and hind wings; (3) forewing MA stem absent; (4) RP + MA originating near wing base; (5) four or eight presectoral crossveins present in both fore- and hind wings; (6) forewing MP deeply bifurcated.

Remarks. This family currently comprises *Cratosmylus* with only one species and *Araripenymphes* with two species. It shares a number of characters with Babinskaiidae, such as the well-developed trichosors, as well as the presence of many presectoral crossveins in both fore- and hind wings. However, it is distinguished from Babinskaiidae by the position of the origin of RP + MA, which is closer to the wing base. In addition, it differs from most babinskaiids except *Paradoxoleon* gen. nov. by the deeply forked forewing MP. Nevertheless, the present phylogenetic analysis did not recover the monophyly of Cratosmylidae, and its phylogenetic status may not be validated, possibly being a synonym of Babinskaiidae. However, considering the deficient knowledge on the morphology of Cratosmylidae due to the scarce fossil records, we temporarily consider it a valid family of Myrmeleontoidea until its phylogenetic position is thoroughly resolved basing on comprehensive morphological evidence.

Genus *Araripenymphes* Menon, Martins-Neto and Martill, 2005

(Figure 1)

Type species: *Araripenymphes seldeni* Menon, Martins-Neto and Martill, 2005

Revised diagnosis. The genus can be distinguished by a combination of the following characters: (1) forewings slenderly elongated, about three times as long as wide (wings broadly ovaid, about two times as long as wide in *Cratosmylus*); (2) forewing costal space rather narrow (much broader in *Cratosmylus*); (3) seven or eight presectoral crossveins present in fore- and hind wings (four presectoral crossveins in fore- and hind wings of *Cratosmylus*); (4) forewing MP branches nearly straight (forewing MP branches distinctly curved posteriad in *Cratosmylus*); (5) forewing CuA distally fused with MP2 (unknown in *Cratosmylus*); (6) hind wing CuA branches very short (hind wing CuA branches much longer in *Cratosmylus*).

*Araripenymphpes burmanus* sp. nov.

LSID: urn:lsid:zoobank.org:act:99D6F1EE-53DD-42BD-A816-2902AC62A77B 

(Figure 1)

Diagnosis. Mostly the same for the genus. In addition, several broad dark markings present on both fore- and hind wings, being located at RP + MA stem, base of RP1, distal MA and MP, and fusing point of MP2 and CuA in forewing, and at distal section of M in hind wing.

Description. Body not preserved. Forewing: Preserved part 18.7 mm long, and 4.5 mm wide; one broad dark marking present at RP + MA stem, and three smaller dark markings present at base of RP1, distal MA and MP, and fusing point of MP2 and CuA; costal space proximally very narrow, slightly broadened distad, around 3.0 times as wide as subcostal space, with 15 simple crossveins preserved; subcostal crossvein not discernible; eight presectoral crossveins present; RP + MA originated from R nearly at proximal 1/5; three rp-ma crossveins present between stem of RP and MA; RP branches mostly not preserved; preserved part of MA simple; six radial crossveins present along stem of RP + MA; MP long, bifurcated at its proximal 1/3, MP1 with at least three branches distally, MP2 fused with CuA at its distal 1/4; 14 crossveins present between MP and CuA; CuA and CuP diverging near wing base; CuA pectinately branched, with at least 20 branches; CuP short, with seven pectinate, short and simple branches; seven cua-cup crossveins present; A1 rather short, connected with CuP with a short crossvein.

Hind wing: Preserved part 19.6 mm long; broad dark markings present on distal portion of wing; single trichosor present between veins along distal margin; costal space narrow, almost as wide as subcostal space, proximal part preserved with 15 simple crossveins; RP + MA originating almost at the same level as that of forewing; eight presectoral crossveins present; MA branched with at least four pectinate branches; MP1 and MP2 straight and long, MP1 distally branched with five pectinate branches, most of them bearing a marginal fork, MP2 pectinately branched at proximal 1/4, with 28 branches, nearly a half of them bearing a marginal fork; CuA pectinately branched, with 10 simple short branches.

Etymology. The specific epithet “*burmanus*” refers to occurrence of the new species in mid-Cretaceous Myanmar amber.

Type material. Holotype: NIGP180156. Amber piece preserving an incomplete adult of *Araripenymphes burmanus* sp. nov. It is polished in the form of an irregular cabochon, clear and transparent, with length × width 27.5 mm × 8.5 mm, height 13.0 mm.

**Figure 2:**
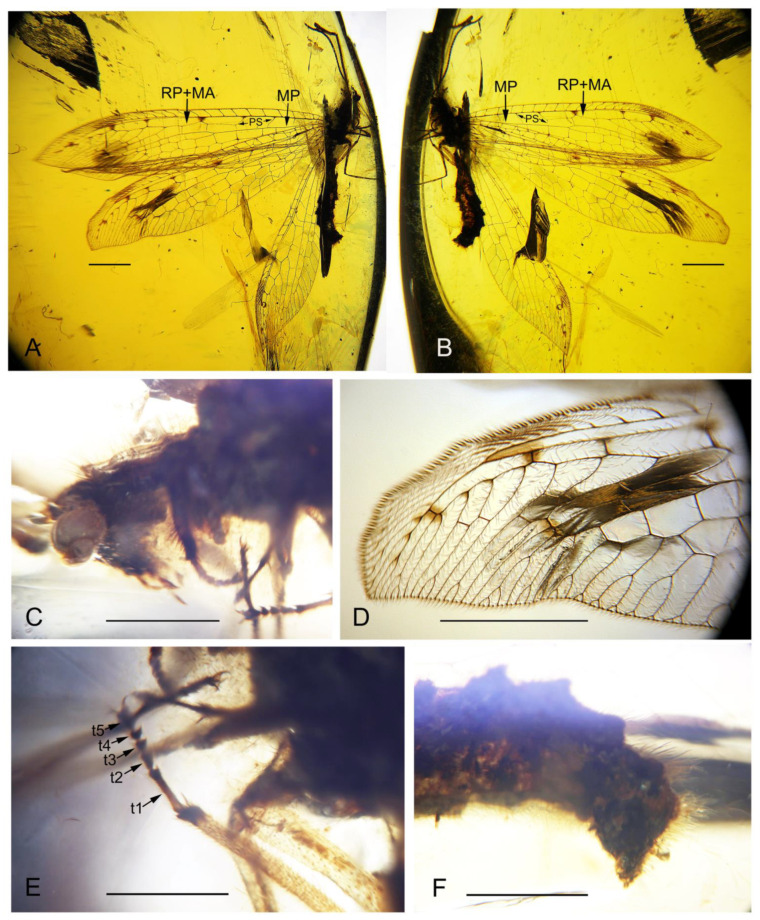
*Paradoxoleon chenruii* gen. et sp. nov., holotype: STJ-703. (**A**) Habitus, right lateral view; (**B**) habitus, left lateral view; (**C**) head and prothorax; (**D**) distal part of right forewing; (**E**) legs; (**F**) female genitalia. Abbreviation: t, tarsomere. Scale bar = 1.0 mm (**A**–**D**); 0.5 mm (**E**,**F**).

**Figure 3:**
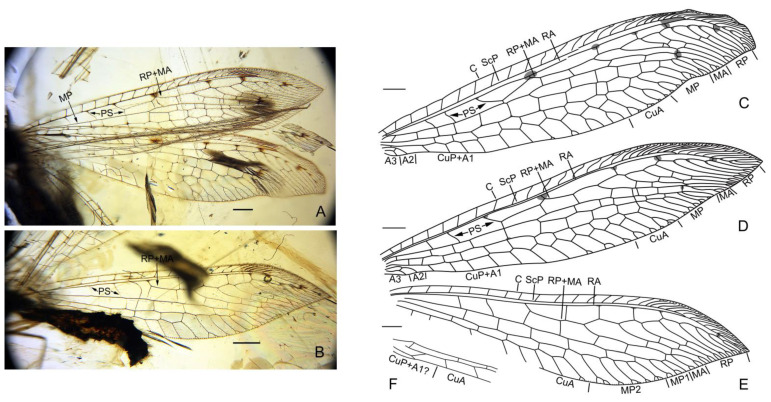
*Paradoxoleon chenruii* gen. et sp. nov., holotype: STJ-703. (**A**,**B**) Photographs of wings: (**A**) wings; (**B**) right hind wing; (**C**–**F**) drawings of wings: (**C**) left forewing; (**D**) right forewing; (**E**) right hind wing; (**F**) left hind wing. Scale bar = 1.0 mm.

Remarks. The new species is undoubtedly placed in *Araripenymphes* by the narrowly elongated wings, the presence of seven or eight presectoral crossveins in both fore- and hind wings, the deeply forked forewing MP, and the distal fusion between forewing MP2 with CuA. However, it differs from *A. seldeni* (the genus type) by the different wing markings. In the new species, the dark markings on forewing are widely separated, and that on hind wing is only present on distal portion. In *A. seldeni*, the dark markings are much broader, almost covering the distal half of forewing and proximal half of hind wing (this feature is distinctly visible in the additional specimen described by Myskowiak et al. ([34]: figure 1). Although it is not described in the holotype, the similar wing markings are probably present in terms of the unevenly colored veins ([13]: figure 1). Moreover, the new species can be distinguished from *A. seldeni* by the short branches on distal part of hind wing MP2. In the latter species, the distal MP2 branches are much longer ([13]: figure 3), ([34]: figure 2).

Family Babinskaiidae Martins-Neto and Vulcano, 1989

Type genus: *Babinskaia* Martins-Neto and Vulcano, 1989

Genus *Paradoxoleon* gen. nov.

LSID: urn:lsid:zoobank.org:act:F4A7C158-0399-4BA3-B427-9AAAB27E5DCE 

(Figure 2 and Figure 3)

Type species: *Paradoxoleon chenruii* sp. nov.

Diagnosis. The new genus can be distinguished from the other genera of Babinskaiidae by a combination of the following characters: (1) three presectoral crossveins present in forewing (4–19 presectoral crossveins in the other genera of Babinskaiidae); (2) forewing distal-most presectoral crossvein distinctly inclined at an angle of 30° with R, and interlinked by a long, longitudinal veinlet with RP + MA (forewing distal-most presectoral crossvein not distinctly inclined, but interlinked by a short longitudinal veinlet with RP + MA in *Burmobabinskaia* Lu, Zhang and Liu, 2017; *Electrobabinskaia* Lu, Zhang and Liu, 2017; and *Gigantobabinskaia* Makarkin and Staniczek, 2019); (3) MP deeply forked proximal to its midpoint (MP simple in the other genera of Babinskaiidae); (4) forewing A1 fused with CuP (shared by *Pseudoneliana* Huang, André and Dany, 2019; *Calobabinskaia* Lu, Wang and Liu, 2021; *Stenobabinskaia* Lu, Wang and Liu, 2021; and *Xiaobabinskaia* Lu, Wang and Liu, 2021; separated from each other in the other genera with preserved anal veins).

Etymology. From “*paradoxo*-” (Latin, meaning “strange”) and “*leon*” (a common genus name of Myrmeleontoidea), in reference to the presence of wing characters that are different from the other genera of Babinskaiidae. Gender: feminine.

Remarks. The new genus is placed in Babinskaiidae on the basis of the filiform antennae, the presence of trichosors, the presence of presectoral crossveins in both fore- and hind wings, and the RP + MA originating nearly at the midpoint of the wing. However, it differs from the other genera of Babinskaiidae by the presence of deeply forked forewing MP and the peculiar configuration of the distal-most presectoral crossvein in the forewing.

*Paradoxoleon chenruii* sp. nov.

LSID: urn:lsid:zoobank.org:act:FE6D7D4D-9658-4F94-8068-792B85601F1D 

(Figure 2 and Figure 3)

Diagnosis. Same as for the genus.

Description. Body length 7.9 mm; head length 0.5 mm; antenna length 4.7 mm; diameter of compound eye 0.5 mm; prothorax length 0.9 mm, mesothorax length 0.7 mm, metathorax length 0.8 mm; forewing 15.1 mm long, 3.4 mm wide; hind wing 12.1 mm long, 2.7 mm wide; abdomen length 5.1 mm.

Head orthognathous, subtriangular. Compound eyes large, semi-globular; antenna filiform, over half of forewing length, slightly narrowed distad, with a sharp tip; scape stout and short, pedicel slender and long, flagellomere shorter and more slender than scape and pedicel, slightly longer than wide. Mouthparts chewing mandibulate.

Prothorax longer than wide, with short setae; meso- and metathorax robust. Legs slender, with sparse short setae; a pair of tibial spurs present, short; tarsus 5-segmented; tarsomere 1 longest, slightly longer than combined length of tarsomeres 2–4; tarsomere 2 twice as long as tarsomere 3 or 4; tarsomere 3 or 4 shortest; tarsomere 5 almost as long as tarsomere 2; pretarsal claws curved, equal in length and shape.

Wings slenderly elongated, around 4.5 times as long as wide; transparent with several dark spots mostly present along the radial space, and also at distal-most subcostal space and branching point of MA; single trichosor present between veins along distal margin in both fore- and hindwings.

Forewing: Costal space slightly broadened distad, around 3.0 times as wide as subcostal space, and nearly as wide as radial space, with 14 simple crossveins on proximal 3/5, and 18 distally bifurcated crossveins and veinlets of ScP + RA on distal 2/5; subcostal crossvein absent; three presectoral crossveins present, with the distal-most presectoral crossvein distinctly inclined at an angle of 30° with R, and interlinked by a long longitudinal veinlet with RP + MA; RP + MA originated from R proximal to midpoint of wing; RP with seven branches in right forewing and eight branches in left forewing, each bearing a marginal fork except RP2 in left forewing; RP7 partially fused with RP8 in left forewing; two incomplete gradate series of crossveins present; MA bifurcated at its distal 1/3, with secondary branches on posterior branch; MP long, diverged into two main branches proximal to midpoint, MP1 and MP2 pectinately branched distally, MP1 with three branches and MP2 with four branches in right forewing, and MP1 with two branches and MP2 with three branches in left forewing; 10–11 crossveins present between MP and CuA; CuA and CuP diverging near wing base; CuA pectinately branched, with three to four branches, each bearing a marginal fork; CuP + A1? long, with four pectinate, short and simple branches; A1 fused with CuP, A2 simple, connected with CuP + A1 by a short crossvein, A3 short and simple, connected with A2 by a short crossvein.

Hind wing: Costal space slightly widened distad, around two times as wide as subcostal space, and distinctly narrower than radial space, with 15 simple crossveins on proximal 2/3, and 14 bufurcated crossveins and veinlets of ScP + RA on distal 1/3; subcostal crossvein absent; two presectoral crossveins preserved; RP + MA originated from R proximal to midpoint of wing; RP with eight branches, each bearing a marginal fork; two gradate series of crossveins present; MA bifurcated at its distal 1/3, each with secondary branch; MP long, diverged into two main branches at wing base, MP1 and MP2 pectinately branched at distal 1/5, MP1 with three branches and MP2 with five branches, mostly bearing a marginal fork; at least six crossveins present between MP and CuA; base of Cu not preserved; CuA pectinately branched, with at least 10 pectinate branches; CuP + A1? short, connected with CuA by a short crossvein.

Abdomen slenderly elongated, segments 1–3 slender, segment 4–6 gradually broadened, segments 7–9 gradually narrowed. Genitalia: Tergum 8 short and narrow; no distinct gonocoxites 8 discernible; tergum 9 slightly shorter than tergum 8; gonocoxites 9 hardly visible; ectoprocts small.

Etymology. The specific epithet “*chenruii*” refers to Dr. Rui Chen, who kindly offered the specimen of this new species for our study.

Type material. Holotype: STJ-703. Amber piece preserving a nearly complete adult of *Paradoxoleon chenruii* gen. et sp. nov. It is polished in the form of a flattened elliptical cabochon, clear and transparent, with length × width 51.8 × 26.8 mm, height 13.0 mm.

### 3.2. Phylogenetic Analysis

**Figure 4:**
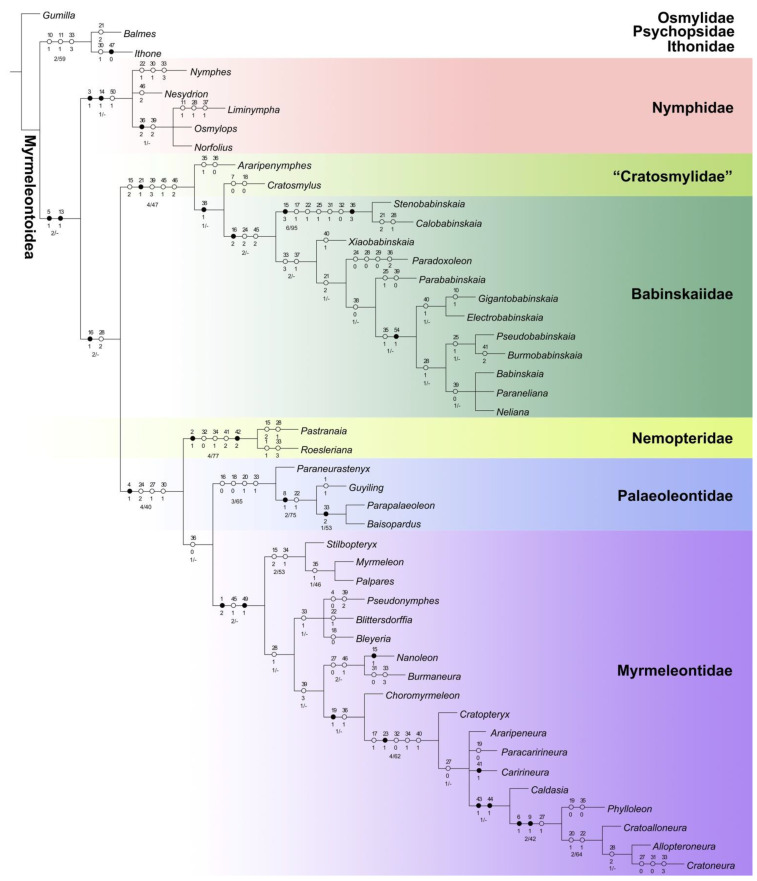
Phylogeny and evolutionary chronogram of extinct and extant Myrmeleontoidea. Topology represents the strict consensus tree of the two most parsimonious trees yielded from TNT. Unambiguous state changes of the morphological characters at the familial and higher level are shown on the tree. Black circle represents the homologous state and white circle represents the homoplasious state. Bremer support values/bootstrap values are shown at relevant nodes.

The maximum parsimony analysis in TNT yielded two most parsimonious trees (MPTs) (length = 162, consistency index = 43, retention index = 80). The strict consensus tree is shown in Figure 4. The present result is largely consistent with that of Lu et al. [18] in the topology, as well as the character states supporting the phylogeny. The major differences refer to the phylogenetic positions of the two genera of Cratosmylidae and some genera of Babinskaiidae. The cratosmylid genera did not form a monophylum, while *Cratosmylus* was found to be a sister to Babinskaiidae on the basis of the forewing CuP fused with A1 (character 38:1). Within Babinskaiidae, the new genus *Paradoxoleon* gen. nov. was recovered to be a sister to the clade including *Parababinskaia* Makarkin, Heads and Wedmann, 2017; *Gigantobabinskaia*; *Electrobabinskaia*; *Pseudobabinskaia* Makarkin, Heads and Wedmann, 2017; *Burmobabinskaia*; *Babinskaia*; *Paraneliana*; and *Neliana* Martins-Neto and Vulcano, 1989, based on the reduction of crossveins in the forewing radial space (21:2). *Paraneliana* herein newly added was assigned into a monophyletic group together with *Babinskaia* and *Neliana*, supported by the forewing A1 shallowly bifurcated (39:0). This lineage was recovered to be sister to the lineage of *Burmobabinskaia* + *Pseudobabinskaia* according to forewing CuA with 5–9 branches (28:1). *Electrobabinskaia* and *Gigantobabinskaia* formed a sister lineage to the monophylum composed of the aforementioned two lineages, according to forewing CuA2 origin proximal to the origin of RP + MA (35:1), and female sternum 6 posteriorly with elongated processes (54:1).

## 4. Discussion

### 4.1. Familial Status of Cratosmylidae

Cratosmylidae is among the extinct myrmeleontoid familes with questionable familial status, possibly being paraphyletic or a stem group in Myrmeleontoidae [23,35]. The two cratosmylid genera *Araripenymphes* and *Cratosmylus* were once placed within the family Nymphidae, and the latter genus was originally placed within the family Osmylidae [13,26,36]. The close relationship of Cratosmylidae and Babinskaiidae was first proposed by Makarkin et al. [23], but no argument was given. The monophyly of Cratosmylidae + Babinskaiidae was first recovered by phylogenetic analysis in Lu et al. [18] and corroborated again in this study. However, the monophyly of Cratosmylidae was herein not supported. Considering the sister-group relationship between *Cratosmylus* and Babinskaiidae, it is likely that the former genus may be a member of Babinskaiidae but not represent a different family because this genus lacks sufficient characters that distinctly differ from Babinskaiidae. The broad forewing (7:0) and short forewing hypostigmal cell (18:0), which were assigned to be autapomorphies of *Cratosmylus*, show weak evidence to support its independent familial status. The other cratosmylid genus *Araripenymphes* appears to be more distantly related to the babinskaiid genera, but it is still hard to consider a separate family because the autapomorphy recovered for this genus, i.e., the forewing CuA2 originating proximal to origin of RP + MA (35:1), is also present in some babinskaiids and antlions [25]. Therefore, the reasonable treatment of Cratosmylidae based on the present result may be the consideration of this family as a junior synonym of Babinskaiidae. Nevertheless, due to the scarcity of cratosmylid fossils, formal taxonomic change of Cratosmylidae should be made until the complete morphological data of this family is available by future findings of more fossils.

### 4.2. New Characters of Babinskaiidae

The morphological diversity of Babinskaiidae, especially those from the Burmese amber, was previously documented in Lu et al. [25]. In light of the new babinskaiid genus herein described, two characters newly found in Babinskaiidae are remarkable. First, there is a longitudinal long veinlet interlinking the distal-most presectoral crossvein and the stem of RP + MA in the forewing of *Paradoxoleon* gen. nov. Interestingly, the distal-most presectoral crossvein is distinctly inclined distad and seems as if it is the stem of RP + MA. Nevertheless, this configuration may be just a highly derived condition from that shared with the babinskaiid genera *Burmobabinskaia*, *Electrobabinskaia*, and *Gigantobabinskaia* ([25]: figures 3 and 5), ([37]: figure 4).

Second, the deeply forked forewing MP with two free branches, which is present in *Paradoxoleon* gen. nov., is first found in Babinskaiidae. This character state is plesiomorphic in Neuroptera and present in most lacewing families except Coniopterygidae (probably due to venational reduction associated with miniaturization) and the myrmeleontoid families Nemopteridae, Palaeoleontidae, and Myrmeleontidae (due to the fusion of MP2 and CuA) [38]. However, the branching form of forewing MP is variable in Nymphidae, being either deeply forked with two long branches in most genera, especially the fossil genera, or unbranched in *Austronymphes* Esben-Petersen, 1914; *Myiodactylus* Brauer, 1866, *Umbranymphes* New, 1984; and some species of *Nymphes* Leach, 1814. Thus, it is not surprising that such variable forewing MP is present also in Babinskaiidae. Moreover, this character state further obscures the interfamilial boundary between Babinskaiidae and Cratosmylidae, which also has the deeply forked forewing MP.

### 4.3. Biogeographical Consideration

The Gondwanan origin of the neuropteridan paleofauna from the mid-Cretaceous of Myanmar has been explored by increasing supportive evidence from Myrmeleontoidea [15,17,24]. The Burmese amber occurrence of *Araripenymphes*, which was only known from the Crato Formation of Brazil, provides important data to verify this hypothesis again. One may question why such ancient biogeographical connection is repeatedly found in Myrmeleontoidea but has not been reported in the other neuropteridan groups. On one hand, the lacewings other than mymeleontoids from the Burmese amber with morphological specializations have not been found from the Lower Cretaceous of Brazil, probably because of their smaller body size and poor preservation in compression fossils may prevent the exploration of these species. On the other hand, the iconic characters of Myrmeleontoidea that are helpful for identification are mainly from the wings, while those of other neuropterans are usually from the head, legs, genitalia, etc. (those to be preserved more difficultly than wings in fossils), which blocks the link between the fossils of other lacewing groups, respectively, from the Kachin amber of Myanmar and the Lower Cretaceous of Brazil through morphological identification. Nevertheless, taking Myrmeleontoidea as an example, such paleofaunal similarity could have existed in broader lineages of Neuropterida, which awaits further investigation.

## 5. Conclusions

The myrmelontoid family Cratosmylidae and Babinskaiidae hitherto only known from the Cretaceous, have rare paleodiversity and obscure phylogenetic position. Our findings provide new morphological evidence for understanding the paleodiversity, historical biogeography, and phylogenetic position of this lineage. The first finding of a deeply bifurcated forewing MP with two free branches in Babinskaiidae highlights the morphologically diversity of this extinct family. The first record of Cratosmylidae from the Kachin amber suggests a Gondwanan origin of the mid-Cretaceous lacewing paleofauna from northern Myanmar. The phylogenetic analysis indicates that Cratosmylidae is probably a junior synonym of Babinskaiidae, but formal taxonomic changes should be made until more complete fossils of this group are available. This study sheds light on the Cretaceous radiation of Neuroptera.

## Data Availability

Data are contained within the article or the Supplementary Materials.

## Supplementary Materials

The following are available online at https://www.mdpi.com/article/10.3390/insects13050429/s1, Table S1. Checklist of Babinskaiidae and Cratosmylidae. Note S1. Morphological character matrix used in the phylogenetic analysis. Table S2. List of characters coded for the phylogenetic analysis. Refs. [39,40,41,42,43,44] are cited in Supplementary Materials.
