# Supplementary material for: New Cretaceous Lacewings in a Transitional Lineage of Myrmeleontoidea and Their Phylogenetic Implications
Source: Insects. 2022 May 5;13(5):429. doi: 10.3390/insects13050429 (PMC9145227; doi:10.3390/insects13050429)
Supplement: Supplementary file 1 [file insects-13-00429-s001.zip › insects-1693751-supplementary.pdf]

Supplementary material for:

New Cretaceous lacewings in a transitional lineage of Myrmeleontoidea and their phylogenetic implications

Xiumei Lu[1], Chunpeng Xu[2,3], Xingyue Liu[4]*

Contents

Table S1. Checklist of Babinskaiidae and Cratosmylidae.

Note S1. Morphological character matrix used in the phylogenetic analysis.

Table S2. List of characters coded for the phylogenetic analysis.

Table S1. Checklist of Babinskaiidae and Cratosmylidae.

**Family Babinskaiidae Martins-Neto & Vulcano, 1989**			
**No.**	**Species**	**Age**	**Locality**
1	*Baisonelia vitimica* Ponomarenko, 1992	Lower Cretaceous, Barremian	Zaza Formation, Russia
2	*Babinskaia formosa* Martins-Neto & Vulcano, 1989	Lower Cretaceous, Upper Aptian	Crato Formation, Brazil
3	*Babinskaia pulchra* Martins-Neto & Vulcano, 1989	Lower Cretaceous, Upper Aptian	Crato Formation, Brazil
4	*Neliana maculata* (Martins-Neto & Vulcano, 1989)	Lower Cretaceous, Upper Aptian	Crato Formation, Brazil
5	*Neliana impolluta* Martins-Neto, 1997	Lower Cretaceous, Upper Aptian	Crato Formation, Brazil
6	*Parababinskaia elegans* Makarkin, Heads &	Lower Cretaceous, Upper Aptian	Crato Formation, Brazil

	Wedmann, 2017		
7	*Burmobabinskaia tenuis* Lu, Zhang & Liu, 2017	mid-Cretaceous, lowest Cenomanian	Tanai, Myitkyina, Kachin, Myanmar
8	*Calobabinskaia xiai* Lu, Wang & Liu, 2021	mid-Cretaceous, Lowest Cenomanian	Tanai, Myitkyina, Kachin, Myanmar
9	*Electrobabinskaia burmana* Lu, Zhang & Liu, 2017	mid-Cretaceous, Lowest Cenomanian	Tanai, Myitkyina, Kachin, Myanmar
10	*Gigantobabinskaia godunkoi* Makarkin & Staniczek, 2019	mid-Cretaceous, Lowest Cenomanian	Tanai, Myitkyina, Kachin, Myanmar
11	*Stenobabinskaia punctata* Lu, Wang & Liu, 2021	mid-Cretaceous, Lowest Cenomanian	Tanai, Myitkyina, Kachin, Myanmar
12	*Parababinskaia makarkini* Hu et al., 2018	mid-Cretaceous, Lowest Cenomanian	Tanai, Myitkyina, Kachin, Myanmar
13	*Parababinskaia douteaui* Ngô-Muller et al., 2020	mid-Cretaceous, Lowest Cenomanian	Tanai, Myitkyina, Kachin, Myanmar
14	*Pseudobabinskaia martinsnetoi* (Lu, Zhang & Liu, 2017)	mid-Cretaceous, Lowest Cenomanian	Tanai, Myitkyina, Kachin, Myanmar
15	*Pseudoneliana makarkini* Huang, André & Dany, 2019	mid-Cretaceous, Lowest Cenomanian	Tanai, Myitkyina, Kachin, Myanmar
16	*Xiaobabinskaia lepidotricha* Lu, Wang & Liu, 2021	mid-Cretaceous, Lowest Cenomanian	Tanai, Myitkyina, Kachin, Myanmar
17	*Paraneliana sennlaubi* Jouault & Nel, 2021	mid-Cretaceous, Lowest Cenomanian	Tanai, Myitkyina, Kachin, Myanmar
18	*Paradoxoleon chenruii* gen. et sp. nov.	mid-Cretaceous, Lowest Cenomanian	Tanai, Myitkyina, Kachin, Myanmar

Family Cratosmylidae Makarkin, Heads & Wedmann, 2017			
**No.**	**Species**	**Age**	**Locality**
1	*Araripenymphes seldeni* Menon, Martins-Neto & Martill, 2005	Lower Cretaceous, Upper Aptian	Crato Formation, Brazil
2	*Cratosmylus magnificus* Myskowiak, Escuillié & Nel, 2015	Lower Cretaceous, Upper Aptian	Crato Formation, Brazil
3	*Araripenymphes burmanus* sp. nov.	mid-Cretaceous, Lowest Cenomanian	Tanai, Myitkyina, Kachin, Myanmar

Note S1. List of characters coded for the phylogenetic analysis.

1. Antenna: (0) filiform, not dilated distad ([39]: Fig. 10g5); (1) filiform, but slightly dilated distad ([39]: Fig. 10g1); (2) distinctly clubbed ([39]: Fig. 10g2).

2. Head with prolonged rostrum: (0) absent ([25]: Fig. 2b); (1) present ([40]: Figs. 1898–1899).

3. Prothorax: (0) not elongated anterior to procoxae ([41]: Figs. 13–20); (1) elongated anterior to procoxae ([14]: Fig. 3).

4. Trichosors: (0) present ([18]: Figs. 2, 5, 7); (1) largely reduced or absent ([18]: Fig. 10f).

5. Nygmata: (0) present; (1) absent ([18]: Fig. 10).

6. Forewing shape: (0) distally not strongly narrowed ([18]: Fig. 10a–e); (1) distally strongly narrowed ([18]: Fig. 10f).

7. Forewing: (0) 2.0 times as long as wide ([42]: Fig. 48); (1) slightly longer than wide ([41]: Figs. 13–20); (2) at least 3.0 times as long as wide ([18]: Fig. 10).

8. Crossveins: (0) normal ([18]: Fig. 10a–e); (2) dense ([18]: Fig. 10e).

9. Forewing costal space: (0) not strongly narrowed proximally ([18]: Fig. 10a–e); (1) strongly narrowed proximally ([18]: Fig. 10f).

10. Forewing costal crossveins on proximal part of costal space: (0) simple ([18]: Fig. 10); (2) forked ([42]: Fig. 48).

11. Forewing humeral veinlet: (0) simple ([18]: Fig. 10); (0) recurrent and branched ([42]: Fig. 48).

12. Forewing Sc and RA: (0) not fused distally ([42]: Fig. 48); (1) fused distally ([18]: Fig. 10); (2) allied together with RP into a triplica ([41]: Figs. 13–20).

13. Forewing ScP and RA terminating: (0) anteriad wing apex ([42]: Fig. 48); (1) at or posteriad wing apex ([18]: Fig. 10).

14. Forewing thyridiate crossveins: (0) absent ([18]: Fig. 10b–f); (1) present ([18]: Fig. 10a).

15. Forewing presectoral crossveins: (0) absent ([18]: Fig. 10a, e–f); (1) present, only one ([15]: Fig. 10); (2) present, more than 2 ([18]: Fig. 10b–d); (3) present, more than 10 ([18]: Figs. 2, 5).

16. Forewing RP+MA: (0) diverging almost from wing base ([18]: Fig. 10a, e); (1) diverging slightly distad wing base ([18]: Fig. 10b, d, f); (2) diverging from a position apparently distad wing base ([18]: Figs. 2, 5, 7, 10c).

17. Forewing prehypostigmal cell: (0) rectangular ([18]: Fig. 10a–e); (1) trapezoidal ([18]: Fig. 10f).

18. Forewing hypostigmal cell: (0) short ([18]: Fig. 10a–b, e); (1) long ([18]: Fig. 10c–d, f).

19. Forewing infra radial cell: (0) short ([18]: Fig. 10); (1) long ([15]: Fig. 2).

20. Forewing branching area of RP+MA: (0) without longitudinally directed outer gradate series of crossveins ([18]: Fig. 10a–d, f); (1) with longitudinally directed outer gradate series of crossveins ([18]: Fig. 10e; 15: Fig. 2).

21. Forewing crossvein of branching area of RP+MA: (0) present throughout this region ([18]: Fig. 10a, d, e–f); (1) absent on distal half of this region ([18]: Fig. 10b); (2). slightly reduced ([18]: Fig. 10c)

22. Forewing with distance between diverging points respectively of MA and RP1: (0) as long as distance between diverging points respectively of RP1 and RP2 ([18]: Fig. 10); (1) at least twice as long as distance between diverging points respectively of RP1 and RP2 ([18]: Figs. 2, 5; [15]: Fig. 2).

23. Forewing MA: (0) dichotomously branched or simple ([18]: Fig. 10a–e); (1) pectinately branched ([18]: Fig. 10f).

24. Forewing MP: (0) deeply forked ([18]: Fig. 10a–b); (1) shallowly forked ([23]: Fig. 8); (2) single ([18]: Fig. 10c–f).

25. Forewing MP1 terminating: (0) far from wing apex ([18]: Fig. 7); (1) near wing apex ([18]: Figs. 2, 5).

26. Forewing single MP branches: (0) dichotomously branched or simple ([18]: Fig. 10d–f); (1) pectinate ([18]: Fig. 10c).

27. Forewing with base of MP2 (oblique vein): (0) absent ([18]: Fig. 10c); (1) present ([18]: Fig. 10d–e; [15]: Fig. 2).

28. Forewing, number of CuA branches: (0) 1–4 ([18]: Fig. 10a); (1) 5–9 ([18]: Fig. 10d); (2) at least 10 ([18]: Fig. 10b–c, e–f). Considering the boundary between CuA and CuP is controversial, the boundary of CuA and CuP in Babinskaiidae follows that in Hu et al (see [24]: Fig. 3A).

29. Forewing CuA branched: (0) at distal 1/3 ([18]: Fig. 10a); (1) near or proximad midpoint ([18]: Fig. 10b–f).

30. Forewing MP2+CuA branching area: (0) not triangular ([18]: Fig. 10a–c); (1) subtriangular ([18]: Fig. 10d–f).

31. Forewing CuA: (0) not forked ([18]: Fig. 10a–c); (1) forked ([18]: Figs. 2, 5, 10d–f).

32. Foweing CuA2: (0) longer than CuA1 branches ([18]: Fig. 10d, f); (1) shorter than CuA1 branches ([18]: Fig. 10e).

33. Forewing CuA2: (0) bifurcated ([18]: Fig. 10d, f); (1) with 3-4 pectinate branches ([35]: Fig. 13); (2) with 6 pectinate branches ([18]: Fig. 10e).

34. Forewing MP1 and MP2+CuA: (0) not closely spaced ([18]: Fig. 10c, e); (1) closely spaced ([18]: Fig. 10d, f).

35. Forewing CuA2 origin: (0) distad origin of RP+MA ([18]: Fig. 10d–f); (1) proximad origin of RP+MA ([15]: Fig. 2).

36. Forewing CuP: (0) short ([18]: Fig. 10b, e); (1) long, terminating near proximal 1/3 of hind margin ([18]: Fig. 10a, c); (2) long, terminating anteriad midpoint of hind margin ([18]: Fig. 10d, f); (3) extremely long, terminating posteriad midpoint of hind margin ([18]: Figs. 2, 5).

37. Forewing CuP: (0) straight ([18]: Fig. 10a–b, d–f); (1) zigzagged ([18]: Figs. 8, 10c).

38. Forewing CuP: (0) not fused with A1 distally ([18]: Fig. 10); (1) fused with A1 distally ([18]: Figs. 2, 5, 7).

39. Forewing A1: (0) shallowly bifurcated ([18]: Fig. 10a, d, f); (1) deeply bifurcated ([42]: Fig. 48); (2) pectinate ([5]: Fig. 109); (3) simple ([18]: Fig. 10c).

40. Hind wing: (0) not strongly tapering distad ([18]: Fig. 10a, b); (1) strongly tapering distad ([18]: Fig. 10c, f).

41. Hind wing: (0) not narrowed ([18]: Fig. 10a, b); (1) slightly narrowed ([15]: Fig. 7); (2) strongly narrowed ([43]: Fig. 1).

42. Hind wing: (0) not elongated, shorter than forewing ([18]: Fig. 10a–c, f); (1) slightly elongated, slightly longer than forewing ([15]: Fig. 7); (2) strongly elongated, apparently longer than forewing ([43]: Fig. 1).

43. Hind wing costal space: (0) narrower than forewing costal space ([18]: Fig. 10a–c); (1) wider than forewing costal space ([18]: Fig. 10f; [15]: Fig. 2).

44. Hind wing costal margin: (0) straight ([18]: Fig. 10a–c); (1) arched ([18]: Fig. 10f).

45. Hind wing RP+MA: (0) diverging almost from wing base ([18]: Fig. 10a); (1) diverging from a position slightly distad wing base ([15]: Fig. 2); (2) diverging from a position near midpoint of wing ([18]: Fig. 10c, f).

46. Hind wing presectorial crossveins: (0) absent ([18]: Fig. 10a); (1) present, only one ([15]: Fig. 10); (2) present, more than 2 ([18]: Fig. 10b–c, f).

47. Hind wing with stem of MA: (0) sigmoid ([42]: Fig. 48); (1) straight or reduced ([18]: Fig. 10a–c, f).

48. Hind wing A2 and A3: (0) preserved ([18]: Fig. 10a); (1) reduced ([18]: Fig. 10c).

49. Tibial spur: (0) small or reduced ([18]: Fig. 1g); (1) long and strout ([15]: Fig. 11).

50. Foreleg arolium: (0) simple ([24]: Fig. 1d); (1) bilobed ([18]: Fig. 1f).

51. Male gonocoxites 9: (0) present as a pair of large external sclerites ([44]: Fig. 3d); (1) present as a pair of relatively small and internal sclerites ([25]: Fig. 2g).

52. Male gonocoxites 9 and gonocoxites 11: (0) not associated tightly into a complex structure ([31]: Figs. 88–89); (1) associated tightly into a complex structure ([31]: Figs. 94–96).

53. Female trichobothria: (0) rosette ([25]: Fig. 4); (1) absent ([15]: Fig. 3).

54. Female sternum 6: (0) posteriorly without elongated processes ([24]: Fig. 7c); (1) posteriorly with elongated processes ([24]: Fig. 7a–b).

Table S2. Morphological character matrix used in the phylogenetic analysis.

No.	Taxon	01	02	03	04	05	06	07	08	09	10	11	12	13	14	15	16	17	18	19	20	21	22	23	24	25	26	27
1	*Gumilla*	0	0	0	0	0	0	2	0	0	0	0	1	0	0	0	0	0	1	0	0	0	0	0	0	0	-	0
2	*Ithone*	0	0	0	0	0	0	0	0	0	1	1	0	0	0	0	0	0	1	0	0	0	0	0	0	0	-	0
3	*Balmes*	0	0	0	0	0	0	1	0	0	1	1	2	0	0	0	0	0	1	0	0	2	0	0	0	0	-	0
4	*Nesydrion*	0	0	1	0	1	0	2	0	0	0	0	1	1	1	0	0	0	1	0	0	0	0	0	0	0	-	0
5	*Nymphes*	0	0	1	0	1	0	2	0	0	0	0	1	1	1	0	0	0	1	0	0	0	1	0	0&1	0	-	0
6	*Norfolius*	0	0	1	0	1	0	2	0	0	0	0	1	1	1	0	0	0	1	0	0	0	0	0	0	0	-	0
7	*Osmylops*	0	0	1	0	1	0	2	0	0	0	0	1	1	1	0	0	0	1	0	0	0	0	0	0	0	-	0
8	*Liminympha*	0	?	1	0	1	0	2	0	0	0	1	1	1	?	0	0	0	1	0	0	0	0	0	0	0	-	0
9	*Cratosmylus*	?	?	0	0	1	0	0	0	0	0	0	1	1	0	2	1	0	0	0	0	1	0	?	0	0	-	0
10	*Araripenymphes*	?	?	0	0	1	0	2	0	0	0	0	1	1	0	2	1	0	?	0	0	1	0	0	0	0	-	0
11	*Paradoxoleon*	0	0	0	0	1	0	2	0	0	?	0	1	1	0	2	2	0	1	0	0	2	0	0	0	0	-	0
12	*Neliana*	0	0	0	0	1	0	2	0	0	0	0	1	1	0	2	2	0	1	0	0	2	0	0	1&2	0	1	0
13	*Paraneliana*	?	0	0	?	1	0	2	0	0	0	0	1	?	0	2	2	0	1	0	0	2	0	0	2	0	1	0
14	*Parababinskaia*	0	0	0	0	1	0	2	0	0	0	0	1	1	0	2	2	0	1	0	0	2	0	0	2	1	1	0
15	*Babinskaia*	0	0	0	0	1	0	2	0	0	0	0	1	1	0	2	2	0	1	0	0	2	0	0	2	0	1	0

16	*Burmobabinskaia*	0	0	0	0	1	?	?	0	0	0	0	?	?	0	2	2	0	?	0	0	2	0	0	2	1	1	0
17	*Electrobabinskaia*	0	0	0	0	1	0	2	0	0	0	0	1	1	0	2	2	0	1	0	0	2	0	0	2	0	1	0
18	*Pseudobabinskaia*	0	0	0	0	1	0	2	0	0	0	0	1	1	0	2	2	0	1	0	0	2	0	0	2	1	1	0
19	*Gigantobabinskaia*	0	?	0	0	1	?	?	0	0	1	0	?	?	0	2	2	?	?	?	?	?	0	?	2	?	1	0
20	*Calobabinskaia*	0	0	0	0	1	0	2	0	0	0	0	1	1	0	3	2	1	1	0	0	2	1	0	2	1	1	0
21	*Stenobabinskaia*	0	0	0	0	1	0	2	0	0	0	0	1	1	0	3	2	1	1	0	0	1	1	0	2	1	1	0
22	*Xiaobabinskaia*	0	0	0	0	1	0	2	0	0	0	0	1	1	0	2	2	0	1	0	0	1	0	0	2	0	1	0
23	*Roesleriana*	1	1	0	1	1	0	2	0	0	0	0	1	1	0	0	1	0	1	0	0	0	0	0	2	0	0	1
24	*Pastranaia*	0	1	0	1	1	0	2	0	0	0	0	1	1	0	2	1	0	1	0	0	0	0	0	2	0	0	1
25	*Choromyrmeleon*	2	0	0	1	1	0	2	0	0	0	0	1	1	0	0	1	0	1	1	0	0	0	0	2	0	0	1
26	*Caririneura*	2	0	0	1	1	0	2	0	0	0	0	1	1	0	0	1	1	1	1	0	0	0	1	2	0	0	0
27	*Cratoneura*	2	0	0	1	1	1	2	0	1	0	0	1	1	0	0	1	1	1	1	1	0	1	1	2	0	0	0
28	*Cratopteryx*	2	0	0	1	1	0	2	0	0	0	0	1	1	0	0	1	1	1	1	0	0	0	1	2	0	0	1
29	*Paracaririneura*	2	0	0	1	1	0	2	0	0	0	0	1	1	0	0	1	1	1	0	0	0	1	1	2	0	0	0
30	*Araripeneura*	2	0	0	1	1	0	2	0	0	0	0	1	1	0	0	1	1	1	1	0	0	1	1	2	0	0	0
31	*Caldasia*	2	0	0	1	1	0	2	0	0	0	0	1	1	0	0	1	1	1	1	0	0	0	1	2	0	0	0
32	*Cratoalloneura*	2	0	0	1	1	1	2	0	1	0	0	1	1	0	0	1	1	1	1	1	0	1	1	2	0	0	1

33	*Allopteroneura*	2	0	0	1	1	1	2	0	1	0	0	1	?	0	0	1	1	1	1	1	0	1	1	2	0	0	1
34	*Phylloleon*	2	0	0	1	1	1	2	0	1	0	0	1	1	0	0	1	1	1	0	0	0	0	1	2	0	0	1
35	*Burmaneura*	2	0	0	1	1	?	2	0	0	0	0	?	?	0	0	1	?	?	?	?	0	?	?	2	0	?	0
36	*Nanoleon*	2	0	0	1	1	0	2	0	0	0	0	1	1	0	1	1	0	1	0	0	0	0	0	2	0	0	0
37	*Bleyeria*	2	0	0	1	1	0	2	0	0	0	0	1	1	0	0	1	0	0	0	0	0	0	0	2	0	0	1
38	*Blittersdorffia*	2	0	0	1	1	0	2	0	0	0	0	1	1	0	0	1	0	1	0	0	0	1	0	2	0	0	1
39	*Pseudonymphes*	2	0	0	0	1	0	2	0	0	0	0	1	1	0	0	1	0	1	0	0	0	0	0	0&2	0	0	1
40	*Palpares*	2	0	0	1	1	0	2	0	0	0	0	1	1	0	2	1	0	1	0	0	0	0	0	2	0	0	1
41	*Stilbopteryx*	2	0	0	1	1	0	2	0	0	0	0	1	1	0	2	1	0	1	0	0	0	0	0	2	0	0	1
42	*Myrmeleon*	2	0	0	1	1	0	2	0	0	0	0	1	1	0	2	1	0	1	0	0	0	0	0	2	0	0	1
43	*Guyiling*	1	0	0	1	1	0	?	1	0	0	0	1	?	0	0	0	0	?	0	1	0	1	?	2	0	0	1
44	*Baisopardus*	0	0	0	1	1	0	2	1	0	0	0	1	1	0	0	0	0	0	0	1	0	1	0	2	0	0	1
45	*Parapalaeoleon*	0	0	0	1	1	0	2	1	0	0	0	1	1	0	0	0	0	0	0	1	0	1	0	2	0	0	1
46	*Paraneurastenyx*	?	?	0	1	1	0	2	0	0	0	0	1	1	0	0	0	0	0	0	1	0	0	?	2	0	0	1

No.	Taxon	28	29	30	31	32	33	34	35	36	37	38	39	40	41	42	43	44	45	46	47	48	49	50	51	52	53	54
1	*Gumilla*	0	0	0	1	0	0	0	0	1	1	0	2	0	0	0	0	0	0	0	1	0	0	0	0	0	0	0
2	*Ithone*	1	1	1	0	0	3	0	0	1	0	0	1	0	0	0	0	0	0	0	0	0	0	0	1	0	0	0
3	*Balmes*	1	0	0	0	1	3	0	0	1	0	0	1	0	0	0	0	0	0	0	1	0	0	0	1	0	0	0
4	*Nesydrion*	0	0	0	0	1	0	0	0	1	0	0	0	0	0	0	0	0	0	2	1	0	0	1	1	0	0	0
5	*Nymphes*	1	1	1	0	1	3	0	0	1	0	0	0	0	0	0	0	0	0	0	1	0	0	1	1	0	0	0
6	*Norfolius*	0	0	0	0	1	0	0	0	2	0	0	2	0	0	0	0	0	0	0	1	0	0	1	1	0	0	0
7	*Osmylops*	0	0	0	0	1	0	0	0	2	0	0	2	0	0	0	0	0	0	0	1	0	0	1	1	0	0	0
8	*Liminympha*	1	0	0	0	1	0	0	0	2	1	0	2	0	0	0	0	0	0	0	1	?	?	?	?	?	?	?
9	*Cratosmylus*	?	1	0	?	?	?	0	0	1	?	1	-	0	0	0	0	0	1	2	1	1	?	?	?	?	?	?
10	*Araripenymphes*	2	1	0	0	1	?	0	1	0	0	0	3	0	0	0	0	0	1	2	1	?	?	?	?	?	?	?
11	*Paradoxoleon*	0	0	0	0	1	3	0	0	2	1	1	3	0	0	0	0	0	2	2	1	1	0	0	?	?	?	?
12	*Neliana*	1&2	1	0	0	1	3	0	1	1	1	0	0	0	0	0	0	0	2	2	1	1	0	?	?	?	?	?
13	*Paraneliana*	1	1	0	0	1	3	0	1	1	1	0	0	0	0	0	0	0	2	2	1	?	?	?	?	?	?	?
14	*Parababinskaia*	1&2	1	0	0	1	3	0	0	1	1	0	0	0	0	0	0	0	2	2	1	1	0	0	1	?	0	0
15	*Babinskaia*	1	1	0	0	1	3	0	1	1	1	0	0	0	0	0	0	0	2	2	1	1	0	?	?	?	?	?
16	*Burmobabinskaia*	?	1	0	0	1	3	0	1	1	1	0	3	?	2	?	0	0	2	2	1	1	0	0	1	?	?	?

17	*Electrobabinskaia*	2	1	0	0	1	3	0	1	1	1	0	3	1	0	0	0	0	2	2	1	1	0	0	1	?	0	1
18	*Pseudobabinskaia*	1	1	0	0	1	3	0	1	1	1	0	3	0	0	0	0	0	2	2	1	1	0	0	?	?	0	1
19	*Gigantobabinskaia*	?	1	?	0	1	3	0	1	1	1	0	3	1	0	0	0	0	2	2	1	1	0	0	?	?	?	?
20	*Calobabinskaia*	1	1	0	1	0	0	0	0	3	0	1	-	0	0	0	0	0	2	2	1	?	0	1	?	?	?	?
21	*Stenobabinskaia*	2	1	0	1	0	0	0	0	3	0	1	-	0	0	0	0	0	2	2	1	1	0	?	1	?	?	?
22	*Xiaobabinskaia*	2	1	0	0	1	3	0	0	1	1	1	-	1	0	0	0	0	2	2	1	1	0	0	?	?	?	0
23	*Roesleriana*	2	1	1	0	0	3	1	0	1	0	?	?	-	2	2	0	0	0	0	1	-	0	0	1	0	0	0
24	*Pastranaia*	1	1	1	1	0	0	1	0	1	0	0	0	-	2	2	0	0	0	0	1	-	0	0	1	0	0	0
25	*Choromyrmeleon*	1	1	1	1	1	0	0	0	1	0	0	3	0	0	0	0	0	1	0	1	?	?	?	?	?	?	?
26	*Caririneura*	1	1	1	1	0	0	1	1	1	0	0	2	1	1	0	0	0	2	2	1	?	?	?	?	?	?	?
27	*Cratoneura*	2	1	1	0	0	3	1	1	1	0	?	?	1	0	0	1	1	1	0	1	?	?	?	?	?	?	?
28	*Cratopteryx*	1	1	1	1	0	0	1	0	1	0	?	?	1	0	0	0	0	1	0	1	?	?	?	?	?	?	?
29	*Paracaririneura*	1	1	1	1	0	0	1	0	1	0	0	3	?	?	?	?	?	?	?	?	?	?	?	?	?	?	?
30	*Araripeneura*	1	1	1	1	0	0	1	1	1	0	0	3	1	0	0	0	0	1	0	1	?	?	?	?	?	?	?
31	*Caldasia*	?	?	?	?	?	?	1	1	?	?	?	?	1	0	0	1	1	?	?	?	?	?	?	?	?	?	?
32	*Cratoalloneura*	1	1	1	1	0	0	1	1	1	0	0	0	1	0	0	1	1	1	0	1	?	?	?	?	?	?	?
33	*Allopteroneura*	2	1	1	1	0	0	1	1	1	0	0	0	1	0	0	1	1	1	0	1	?	1	0	?	?	?	?

34	*Phylloleon*	1&2	1	1	1	0	0	1	0	1	0	0	0	1	0&1	0&1	1	1	2	2	1	0	1	0	?	?	1	0
35	*Burmaneura*	1	1	1	0	1	3	0	0	0	0	0	3	0	0	0	0	0	1	1	1	0	1	?	?	?	?	?
36	*Nanoleon*	1	1	1	1	1	0	0	0	0	0	0	3	0	0	0	0	0	1	1	1	0	1	0	?	?	1	0
37	*Bleyeria*	1	1	1	1	1	1	0	0	0	0	0	0	0	0	0	0	0	1	0	1	?	?	?	?	?	?	?
38	*Blittersdorffia*	1&2	1	1	1	1	1	0	0	0	0	0	0	0	0	0	0	0	1	0	1	?	?	?	?	?	?	?
39	*Pseudonymphes*	0&1	1	1	1	1	1	0	0	0	0	0	2	0	0	0	0	0	1	0	1	0	?	?	?	?	?	?
40	*Palpares*	2	1	1	1	1	0	1	1	0	0	0	0	0	0	0	0	0	1	2	1	0	1	0	1	1	1	0
41	*Stilbopteryx*	2	1	1	1	1	0	1	0	0	0	0	0	0	0	0	0	0	1	1	1	0	1	0	1	1	1	0
42	*Myrmeleon*	2	1	1	1	1	0	1	1	0	0	0	0	0	0	0	0	0	1	2	1	0	1	0	1	1	1	0
43	*Guyiling*	2	1	1	1	1	1	0	0	0	0	?	?	?	?	0	0	0	0	0	1	?	0	?	?	?	?	?
44	*Baisopardus*	2	1	1	1	1	2	0	0	0	0	0	3	0	0	0	0	0	0	0	1	0	0	?	?	?	?	?
45	*Parapalaeoleon*	2	1	1	1	1	2	0	0	0	0	0	3	0	0	?	?	?	?	?	?	?	?	?	?	?	?	?
46	*Paraneurastenyx*	2	1	1	1	1	1	0	0	0	0	0	0	?	?	?	?	?	?	?	?	?	?	?	?	?	?	?
